# Linking Plant Nutritional Status to Plant-Microbe Interactions

**DOI:** 10.1371/journal.pone.0068555

**Published:** 2013-07-16

**Authors:** Lilia C. Carvalhais, Paul G. Dennis, Ben Fan, Dmitri Fedoseyenko, Kinga Kierul, Anke Becker, Nicolaus von Wiren, Rainer Borriss

**Affiliations:** 1 Molecular Plant Nutrition, University of Hohenheim, Stuttgart, Germany; 2 Bakteriengenetik, Institut für Biologie, Humboldt Universität Berlin, Berlin, Germany; 3 Australian Centre for Ecogenomics, School of Chemistry and Molecular Biosciences, The University of Queensland, Brisbane, Queensland, Australia; 4 Advanced Water Management Centre, The University of Queensland, Brisbane, Queensland, Australia; 5 Institute of Forest Protection, Nanjing Forestry University, Nanjing, China; 6 Molecular Plant Nutrition, Leibniz-Institute for Plant Genetics and Crop Plant Research, Gatersleben, Germany; 7 Molekulare Genetik, Institut für Biologie III, Albert-Ludwigs-Universität, Freiburg, Germany; 8 ABiTEP GmbH, Berlin, Germany; United States Department of Agriculture, Agricultural Research Service, United States of America

## Abstract

Plants have developed a wide-range of adaptations to overcome nutrient limitation, including changes to the quantity and composition of carbon-containing compounds released by roots. Root-associated bacteria are largely influenced by these compounds which can be perceived as signals or substrates. Here, we evaluate the effect of root exudates collected from maize plants grown under nitrogen (N), phosphate (P), iron (Fe) and potassium (K) deficiencies on the transcriptome of the plant growth promoting rhizobacterium (PGPR) *Bacillus amyloliquefaciens* FZB42. The largest shifts in gene expression patterns were observed in cells exposed to exudates from N-, followed by P-deficient plants. Exudates from N-deprived maize triggered a general stress response in FZB42 in the exponential growth phase, which was evidenced by the suppression of numerous genes involved in protein synthesis. Exudates from P-deficient plants induced bacterial genes involved in chemotaxis and motility whilst exudates released by Fe and K deficient plants did not cause dramatic changes in the bacterial transcriptome during exponential growth phase. Global transcriptional changes in bacteria elicited by nutrient deficient maize exudates were significantly correlated with concentrations of the amino acids aspartate, valine and glutamate in root exudates suggesting that transcriptional profiling of FZB42 associated with metabolomics of N, P, Fe and K-deficient maize root exudates is a powerful approach to better understand plant-microbe interactions under conditions of nutritional stress.

## Introduction

The release of carbon-containing compounds (rhizodeposits) from plant roots is known to improve plant nutrient acquisition and to influence the diversity and composition of rhizosphere bacterial communities [1]–[3]. Rhizosphere bacteria can affect plant productivity by causing or suppressing disease, by producing plant growth regulators, and other biologically active substances, or by modulating the availability of nutrients and toxic elements [4]. Those that exert beneficial effects on plant productivity are known as plant growth-promoting rhizobacteria (PGPR) [5] and represent a potentially ‘green’ alternative to the intensive use of artificial fertilizers and pesticides in agricultural systems [6], [7]. To date, attempts to use PGPR to increase crop yields have resulted in mixed successes [8], [9]. Improved exploitation of these organisms requires a deeper understanding of how they communicate with their host and how these interactions are influenced by the environment.

While significant progress has been made in understanding how specific compounds, secreted from plant roots, select for microbial populations, the extent to which nutrient deficiencies affect plant-microbe interactions via modified rhizodeposition patterns remains elusive. This information is necessary to facilitate the management of native or introduced microorganisms that improve plant productivity.

Rhizosphere microbial community composition has been shown to differ between plants exposed to different nutrient limitations [10], [11]. These differences are thought to relate to modifications in rhizodeposition patterns. When exposed to nutrient limitations, plants exhibit a wide-range of responses that include changes to the quantity and composition of the rhizodeposits released by roots [12]. In a previous study using maize, we demonstrated that N-deficiency reduced the release of amino acids in root exudates, P deficiency stimulated the release of *gamma*-aminobutyric acid, GABA, and carbohydrates, K-deficient plants released less sugars (particularly glycerol, ribitol, fructose and maltose), and Fe-deficiency increased the release of glutamate, glucose, ribitol and citrate [12].

A number of studies have shown that PGPR can facilitate increased nutrient acquisition by plants experiencing nutrient limitation. For example, I) *Azospirillum lipoferum* strain AZm5 promoted the growth of N-deficient tomato seedlings [13], II) *B. amyloliquefaciens* FZB45 stimulated the growth of P-deficient maize seedlings in the presence of phytate as an organic P source [14], III) *Bacillus edaphicus* increased shoot and root growth of K deficient cotton and rape [15], and IV) *Pseudomonas* strain GRP3A enhanced the growth of Fe-deficient *Vigna radiata* [16]. The interactions that occur between nutrient-deficient plants and PGPR that trigger microbial activities which benefit plants are poorly understood; however, carbon-containing rhizodeposits are thought to play an important role [17].

The effects of root exudates on genome-wide gene expression profiles have been characterized for some PGPR including the model strain for gram-positive PGPR, *Bacillus amyloliquefaciens* FZB42 [18]–[21]. In case of FZB42, 302 genes were identified as being differentially transcribed in presence of maize root exudates [20]. However, these investigations were either performed using exudates collected from plants that were supplied optimal levels of all nutrients, or did not take into consideration the nutritional status of the plants. For this reason, our understanding of how individual nutrient deficiencies influence the interactions between plants and individual PGPR is poor.

In a previous study, we characterized the influence of nutrient deficiencies on maize root exudation and clear differences in the metabolite profiles between treatments were revealed [12]. In the present study, we exposed the PGPR *Bacillus amyloliquefaciens* FZB42 to those exudates and characterized bacterial responses at the transcriptional level. To account for differential responses attributed to the physiological state of bacterial cells, exudate-exposed cultures were harvested in two growth phases: exponential and transient. Finally, we were able to relate changes in bacterial transcript profiles to primary metabolites present in the exudates. By integrating bacterial transcriptomics with root exudate metabolomics, we provide a new insight into plant-microbial communication under conditions of distinct nutrient deficiencies.

## Materials and Methods

The effect of root exudates collected from maize plants deficient in N, P, Fe and K on the transcriptome of *B. amyloliquefaciens* FZB42 was investigated. The transcriptional profiles gained by bacterial populations after incubation with these exudates (treatment) were compared to the ones gained in response to exudates collected from nutrient sufficient plants (control). Bacterial cells incubated with exudates were harvested at two growth phases – exponential (optical density of 1.0 at 600 nm) and transient (optical density of 3.0 at 600 nm), which will hereafter be referred as OD 1.0 and OD 3.0, respectively [20].

### Plant Growth Conditions

Maize seeds (*Zea mays* L. var. Surprise) were shaken for three min in 96% ethanol, 30 min in 3% sodium hypochlorite solution, rinsed twice in sterile distilled water (SDW) and then left to soak in SDW for 4 h at 25°C. Sterility of seeds was confirmed by the absence of microbial growth in liquid Luria-Bertani (LB) and semi-solid Tryptic Soy Agar media (TSA, 0.3% Agar) to which seeds had been added and incubated for seven days at 37°C. Surface sterilized seeds were pre-germinated on solid half-strength Murashige Skoog medium containing 1% sucrose and 0.7% agar (Difco, Becton Dickinson) and maintained at 28°C in the dark. Seedlings were transferred to glass bottles designed to facilitate axenic growth conditions [22]. The hydroponic system was permanently aerated and maintained in a controlled environment chamber at 60% humidity, 8 h darkness at 20°C, and 16 h light at 280 µmol photons m^−2^ s^−1^ and 25°C. The composition of the nutrient solution was as follows: 2.0 mM Ca(NO_3_)_2_, 0.7 mM K_2_SO_4_, 0.5 mM MgSO_4_, 0.1 mM KCl, 0.1 mM KH_2_PO_4_, 1.0 µM H_3_BO_3_, 0.5 µM MnSO_4_, 0.5 µM ZnSO_4_, 0.2 µM CuSO_4_, 0.01 µM (NH_4_)_6_Mo_7_O_24_ and 100 µM Fe(III)-EDTA.

### Root Exudates Collection

Root exudates from nutrient sufficient and N-, P-, Fe- or K-deficient plants were collected as described previously [12]. Briefly, each deficiency was induced by omitting the corresponding nutrient to the nutrient solution. To maintain the ion balance of the nutrient solution, Ca(NO_3_)_2_, KH_2_PO_4_ and K_2_SO_4_ were replaced with CaCl_2_, KCl and MgSO_4_, respectively. The nutrient solution was changed once in the first seven days and then after every time that root exudates were collected. During every nutrient solution replacement, a 100 µL aliquot was withdrawn and spread on solid LB medium to check for sterility. Contaminated vessels were discarded. Root exudates were collected 13, 14 and 15 d post-germination (fourth-leaf stage), pooled within treatment, freeze-dried and then stored at −20°C. Exudates were collected at this developmental stage to ensure that carbon associated with seed reserves was exhausted. Two hours after the onset of the light period the nutrient solution was replaced with autoclaved ultrapure water in which root exudates were collected for 6 h. The root system was aerated throughout the cultivation and collection period to avoid oxygen limitation.

### Chemical Analyses of Root Exudates

The analyses were focused on primary metabolites, namely sugars, amino acids, as well as organic acids and performed as previously described [12]. Amino acids were measured using a Shimadzu high-performance liquid chromatography (HPLC) system equipped with a fluorescence detector. From each sample a 40 µL-aliquot was derivatized by 160 µL OPA (o-phtaldialdehyde) reagent, and 20 µL of the resulting mixture was injected and separated on a GROM-SIL OPA-3 column (3 µm, 125×4.0 mm) using gradient elution by solvent A (25 mM phosphate buffer pH 7.2 with 0.75% tetrahydrofuran) and solvent B (methanol :acetonitrile :25 mM phosphate buffer pH 7.2 (35∶15∶50) (v:v:v)). Gradient profile: 0–2 min, 0% B; 2–10 min, 0–50% B; 10–15 min, 50–60% B; 15–20 min, 60–100% B; 20–25 min, 100% B; 25–26 min, 100-0% B; 26–35 min, 0% B. The flow rate was 1 mL min^−1^. Subsequent fluorescence detection of the derivatives was performed at an excitation wavelength of 330 nm and 450 nm for fluorescence emission. Organic acids were determined by ion chromatography (Dionex, Idstein, Germany) equipped with conductivity detector and suppressor ASRS Ultra II. For each sample a 20 µl volume was separated on the Dionex IonPac AS 11 HC column (2×250 mm) using gradient elution starting from 4 mM KOH (0–4 min), then a stepwise linear increase to 80 mM over 28 min (4–10 min, 4–15 mM; 10–14 min, 15–25 mM; 14–24 min, 25–80 mM; 24–28 min, 80 mM), followed by re-equilibration to 4 mM for 2 min and 10 min equilibration by 4 mM KOH. The flow rate was 0.2 ml min^−1^. Organic acids were identified by comparison of retention time with known standards. Sugars were determined by gas chromatography–time of flight mass spectrometry (GC-TOF-MS) [23]. A lyophilized 75 µL aliquot of root exudates was dissolved in 50 µL methoxyamine hydrochloride in dry pyridine and derivatized for 2 h at 37°C followed by a 30 min treatment with 50 µl N-methyl-N-trifluoroacetamide at 37°C. A volume of 1 µL was injected into the GC column in a splitless mode.

### Incubation of Bacterial Cells with Root Exudates

A starter-culture was prepared by inoculating a single overnight colony of *B. amyloliquefaciens* FZB42 into five mL of the 1C medium (0.7% tryptone, 0.3% peptone, 0.1% glucose, 0.5% NaCl and 0.1% glucose). When cells in the starter-culture reached an optical density of 1.0 at 600 nm (OD), an aliquot was transferred to a new 1C medium up to a final OD of 0.01. The new 1C medium was supplemented with 10% of a soil extract and 250 µg dry weight root exudates per mL immediately before inoculation. A sterile solution referred here as soil extract was incorporated into the media to partially simulate chemical conditions that bacteria experience in soil environments. It was prepared as follows: 1 L deionized water was mixed with 500 g compost soil for 15 min, passed through a filter and then autoclaved for 20 min at 120°C. This soil extract was previously shown to exert minor changes on the transcriptome of FZB42 [20]. Just five genes were repressed, and only one of which was related to plant nutrition (*iolS*, which encodes an inositol utilization protein) [20]. Bacterial cells were incubated at 24°C, 210 rpm and after approximately 8 and 14 h were harvested at OD 1.0 (exponential phase), and OD 3.0 (transient phase), respectively. Root exudates were added into the main culture up to a final concentration of 250 µg of dry weight per mL of culture medium. Three biological replicates were obtained for control bacterial cultures supplemented with root exudates from nutrient replete plants and the treatments supplemented with exudates from nutrient deficient plants. The bacterial culture was mixed to a buffer composed of 20 mM Tris-HCl, 25 mM MgCl_2_ and 20 mM NaN_3_ at a ratio of 1∶2, and then centrifuged at 5,000 rpm at ambient temperature, for 4 min. The supernatant was discarded and the pellet was resuspended in 1 mL of the same buffer, and centrifuged again at 9,000 rpm and 4°C for 4 min. To avoid RNA degradation, only four samples were processed simultaneously, therefore adding and decanting the buffer added a maximum of two minutes. The pellets were then stored at –80°C after being snap frozen in liquid nitrogen.

### RNA Purification, Labeling of cDNA, Microarray Design, Hybridization, Image Acquisition and Microarray Analysis

Total RNA was isolated from 15 mL and 7 mL of bacterial cell cultures at exponential and transient growth phases, respectively, following the manufacturer’s instructions of the RNA purification kit NucleoSpin®RNA L (MACHEREY-NAGEL GmbH & Co.KG, Düren, Germany). Starting from 10 to 30 µg of total RNA, random hexamer primers (Qiagen-Operon, Hilden, Germany), Superscript III RT (Stratagene, La Jolla, CA), and 0.5 mM dNTP, dTTP aminoallyl-dUTP (1∶4, dNTPs, PeqLab, Erlagen, Germany; aa-dUTP: Sigma-Aldrich, Taufkirchen, Germany) were used to synthesize aminoallyl-modified first-strand cDNA by reverse transcription at 42°C for 90 min. After hydrolysis and clean-up using CyScribe GFX purification columns (GE Healthcare, Munich, Germany), Cy3- and Cy5-N-hydroxysuccinimidyl ester dyes (GE Healthcare) were coupled to the aminoallyl-labeled first-strand cDNA. Uncoupled dye was removed using the CyScribe GFX Purification kit. The design of the microarray Bam4kOLI was based on the complete genome sequence of *B. amyloliquefaciens* FZB42 [20], [24]. A number of 3,931 50–70 mer oligonucleotides representing predicted protein-encoding genes and a set of small non-coding RNA were spotted on the array. Oligonucleotides were designed using the Oligo Designer software from Bioinformatics Resource Facility, CeBiTec, Bielefeld University. The microarray included the antibiotic resistance genes *Em^r^, Cm^r^, Nm^r^* and *Spc^r^* as alien DNA oligonucleotides and eight spiking controls as well as one empty control (nothing spotted). These genes encode the antibiotics erythromycin, chloramphenicol, neomycin, and spectinomycin, respectively. Stringency controls with 71, 80, and 89% similarity to native sequences of the genes *dnaA*, *rpsL*, *rpsO*, *rpsP*, and *rpmI* were added to evaluate cross hybridization. Four replicates of each oligonucleotide probe were spotted on each array. Microarrays were prehybridized for 45 min at 42°C in Easyhyb hybridization solution (Roche Diagnostics, Mannheim, Germany) supplemented with 5 µg ml^−1^ sonicated salmon sperm DNA. The Cy3 dye was used to label the cDNA derived from the treatment and the Cy5 to label the cDNA derived from the control. Following prehybridization, microarrays were washed in Milli-Q water (21°C, 1 min), submerged in ethanol (21°C, 10 s) and centrifuged (185×*g*, 3 min, 20°C). Hybridization was performed at 42°C for 16 h in Easyhyb hybridization solution (Roche Diagnostics, Mannheim, Germany) supplemented with 50 µg mL^−1^ sonicated salmon sperm DNA in a final volume of 65 µL under a cover slip. Before applying the hybridization solution to the microarray, it was denatured for 5 min at 65°C. Microarrays were washed once in 2×saline sodium citrate (SSC), 0.2% sodium dodecyl sulfate (SDS, 5 min, 42°C), twice in 0.2×SSC, 0.1% SDS (2 min, 21°C) and twice in 0.2×SSC (2 min, 21°C). Slides were subsequently dried by centrifugation (3 min, 185×*g*, 20°C) and scanned at a pixel size of 10 µm using the ScanArray 4000 microarray scanner (Perkin-Elmer, Boston, MA, USA). Mean signal and mean local background intensities were obtained for each spot of the microarray images using the ImaGene 5.0 software for spot detection, image segmentation and signal quantification (Biodiscovery Inc., Los Angeles, CA, USA). Spots were flagged as “empty” in case *R* ≤1.5, where *R* = (signal mean−background mean)/background standard deviation. The remaining spots were considered for further analysis. The log_2_ of the ratio of intensities (M) was calculated for each spot using the formula *M_i_* = log_2_ (*R_i_*/*G_i_*). *R_i_* = *I*_ch1*i*_−Bg_ch1*i*_ and *G_i_* = *I*_ch2*i*_−Bg_ch2*i*_, where *I*_ch1*i*_ or *I*_ch2*i*_ is the intensity of a spot in channel 1 or channel 2 and Bg_ch1*i*_ or Bg_ch2*i*_ is the background intensity of a spot in channel 1 or channel 2, respectively. The mean intensity was calculated for each spot *A_i_* = log_2_ (*R_i_G_i_*)^0.5^ [25]. The method of LOWESS (Locally Weighted Scattered Plot Smoothing) was used to normalize raw data. Adjusted p-values (also known as q-values) were determined using the False Discovery Rate (FDR) control method. FDR-adjusted p-values of less than 0.05 were used to indicate significance. Genes were considered differentially expressed if their levels of expression differed at least twofold from the control (M ≥0.8 or ≤ − 0.8). Normalization and t-statistics were carried out using the EMMA 2.8.2 software (http://www.genetik.uni-bielefeld.de/emma) [26]. Relative transcript levels from the genes mentioned in the ‘Results and Discussion’ section were depicted as heat maps which were generated using the Genesis software, release 1.7.6 [27].

### Gene Ontology Analyses

The DAVID database was used to evaluate functional enrichment, also known as Gene Ontology (GO) term enrichment analysis (http://david.abcc.ncifcrf.gov/). The background was set to the total list of genes spotted on *B. amyloliquefaciens* FZB42 microarrays for lists of up- and down-regulated genes corresponding to each treatment.

### Multivariate Statistical Analysis of Microarrays Data

A constrained ordination method, known as ‘Between Group Analysis’ (BGA), has been successfully applied to microarray data analysis. This approach allows the investigator to enter information regarding the treatment structure of the experiment and to visualize the maximum variation that can be attributed to the treatment groups [28], [29]. A BGA based on ‘Correspondence Analysis’ (CA) [29] was performed to evaluate whether changes in bacterial transcript levels after incubation with different root exudates can be distinguished and to identify the most discriminating genes for each deficiency treatment. BGA is analogous to Canonical Correspondence Analysis and was implemented using the R package *multistab* [30]. To identify metabolites that correlated with specific genes and treatments, we performed Correspondence Analysis (CA) on the transcript profiles and superimposed fitted vectors of metabolites (z-scores) that correlated significantly with the CA axes. This analysis was implemented using the R package *vegan*.

### Quantitative Real-time PCR

Total RNA (1 µg) was reverse-transcribed with RevertAid™ Premium Reverse Transcriptase (Fermentas, St. Leon-Rot, Germany) according to the manufacturer’s instructions, using random hexamers as primers. The real-time PCR was carried out using 7500 Fast Real-Time PCR System (Carlsbad, California, USA). Each 5 µL reaction consisted of 1 µL template cDNA (1∶10–1∶10000), 500 nM each primer and 2.5 µL SYBR® Green PCR Master Mix (Carlsbad, California, USA). A 40-cycle amplification was performed (95°C for 3 sec, and 60°C for 30 sec). Target cDNA from reference and experimental samples were amplified in triplicate. The length of PCR products ranged from 59 to 80 bp. Normalization of results was performed relative to gene expression levels of *gyrA*, which did not show altered expression under any of the conditions tested in all microarrays. Quantification was based on the analysis of threshold cycle (Ct) values [31]. A table with the sequences of primers used in this study is provided as Table S1.

### Microarray Data Accession Numbers

Complete lists of differentially expressed genes in each treatment combination (nutrient deficiency *versus* growth phase) are documented in the Supplementary online material (Tables S2, S4, S6 and S8). The microarray data are available from the ArrayExpress database (http://www.ebi.ac.uk/arrayexpress/) under the accession number E-MEXP-3795 in the Minimum Information About a Microarray Experiment (MIAME)-compliant format.

## Results and Discussion

### Overall Changes in Gene Expression in Response to Root Exudates

**Figure 1:**
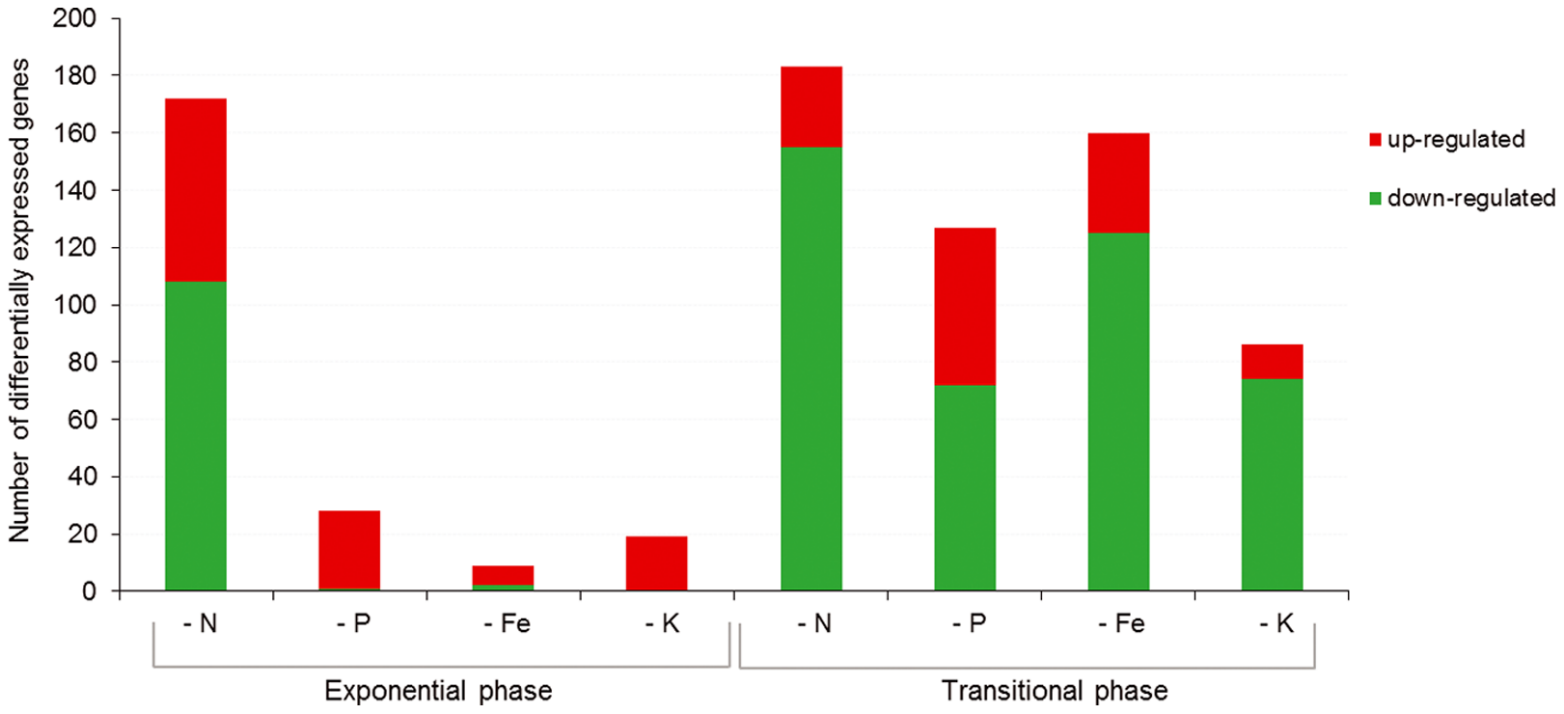
Number of differentially expressed genes of FZB42 in exponential (OD 1.0) and transient (OD 3.0) growth phases in response to nutrient-deficient maize root exudates treatments. ‘-N’ denotes nitrogen deficiency; ‘-P’, phosphorus deficiency, ‘-Fe’, iron deficiency; ‘-K’, potassium deficiency.

In our previous studies we found that adding of root exudates up to a final concentration of 250 mg dry weight per L of culture medium is sufficient to cause a significant response of the FZB42 transcriptome and proteome during transient growth stage [19], [20], [22]. Here, bacterial cells were harvested for RNA extraction in the exponential growth stage [OD_600_ = 1.0], and in the transient growth stage [OD_600_ = 3.0]. Corroborating our previous results, we found that the number of down-regulated genes was dramatically increased in cells harvested during the transient growth stage, with one remarkable exception: treatment with exudates obtained from the maize plants grown under conditions of nitrogen deficiency resulted in a different transcription pattern of more than 170 genes. In general, the number of down-regulated genes of cells harvested during transient growth phase and exposed to root exudates of maize plants grown under nutritional limitation were higher than the number of up-regulated genes when compared with the transcription profile of cells exposed to exudates from maize plants grown without nutritional limitation (Fig. 1).

### Validation of Microarrays

Genes identified as being up- or down-regulated using microarrays were randomly selected to be confirmed using quantitative real time PCR (qRT-PCR, Table 1). Relative to the microarray measurements, however, larger fold-changes were detected using qRT-PCR (Table 1). This finding is consistent with previous comparisons of microarray and real time PCR measurements of transcript abundances [32]–[34].

**Table 1:** List of genes selected for validation of microarray results by real-time RT-PCR.

Treatment	Gene ID	Gene	FC*Microarray	FC Real-timeRT PCR
-Fe	RBAM_035760	*licH*	−6.6	−187.6
-Fe	RBAM_036760	*iolC*	−2.5	−21.3
-Fe	RBAM_030250	*yvqH*	−4.3	−9.6
-Fe	RBAM_019060	*dhaS*	−3.2	−3.6
-Fe	RBAM_035790	*licB*	−3.3	−111.0
-P	RBAM_032550	*flgL*	2.4	2.8
-P	RBAM_032490	*fliS*	2.5	2.7
-P	RBAM0_36710	*iolH*	−2.5	−9.9
-P	RBAM_018960	*yocH*	−3.2	−2.7
-N	RBAM_001110	*clpC*	−2.0	−2.2
-N	RBAM_036710	*iolH*	−4.7	−28.5
-N	RBAM_002320	*glmS*	2.2	2.3
-N	RBAM_018960	*yocH*	−2.0	−1.9
-K	RBAM_019060	*dhaS*	−2.1	−68.6
-K	RBAM_036760	*iolC*	−2.1	−244.0
-K	RBAM_018960	*yocH*	−2.7	−22.1
-K	RBAM_030250	*yvqH*	−5.5	−305.8

Root exudates from the iron deficiency treatment are represented as ‘-Fe’, phosphorus deficiency as ‘-P’, nitrogen deficiency as ‘-N’, potassium deficiency as ‘-K’.

* FC stands for Fold-Change.

### Bacterial Responses to Exudates of N-deficient Plants

**Figure 2:**
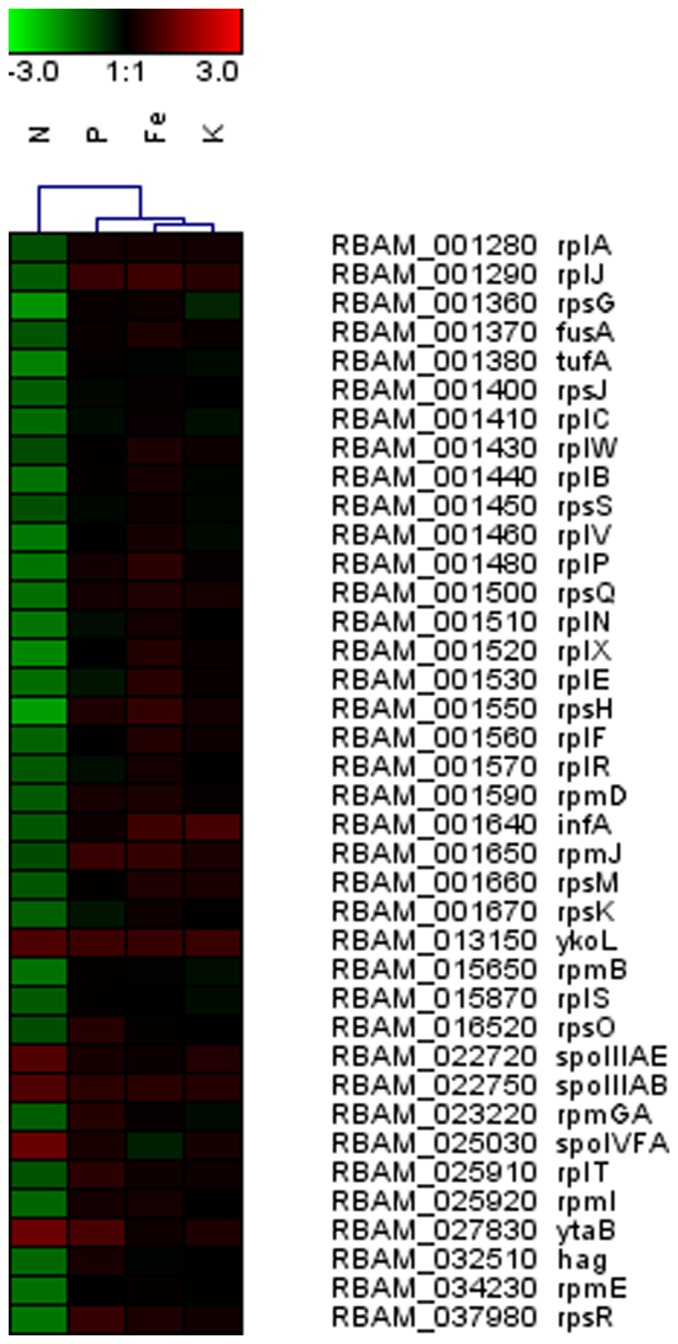
Heat map depicting relative transcript levels of differentially expressed genes in FZB42 exposed to root exudates collected from maize cultivated under nitrogen (N), phosphate (P), iron (Fe), and potassium (K) deficiencies in the exponential growth phase (OD 1.0). Only genes that have been referred in the ‘Results and Discussion’ section are displayed, the full list of differentially expressed genes is shown in the Tables S2, S4, S6, and S8.

**Figure 3:**
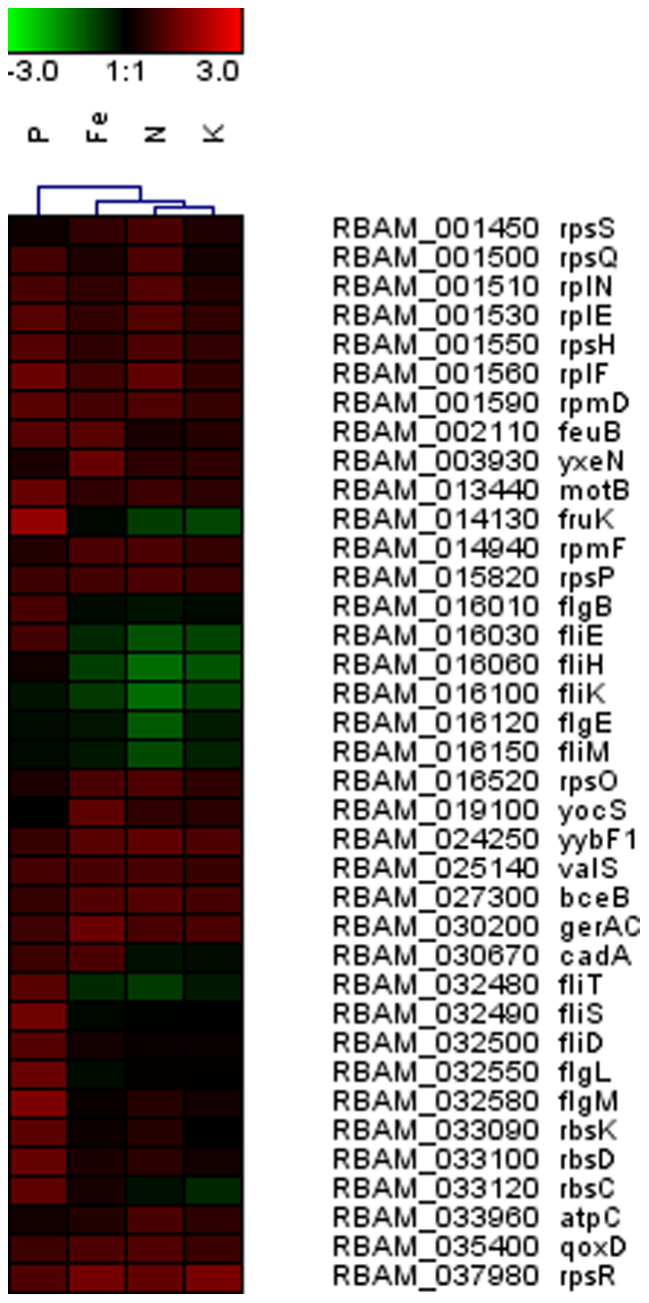
Heat map depicting relative transcript levels of differentially expressed genes in FZB42 exposed to root exudates collected from maize cultivated under nitrogen (N), phosphate (P), iron (Fe), and potassium (K) deficiencies in the transient growth phase (OD 3.0). Only genes that have been referred in in the ‘Results and Discussion’ section are displayed, the full list of differentially expressed genes is shown in the Tables S2, S4, S6, and S8.

Exudates from N-deficient plants elicited differential transcription of stress-related genes in bacterial cells. Out of a total number of 108 genes, 32 genes involved in protein synthesis (ribosomal proteins and translation initiation factors) were down-regulated in the exponential growth phase, whilst no gene involved in translation was found up-regulated in this growth stage (Fig. 2, Tables S2 & S3). When bacteria are exposed to environmental stresses, including amino acid deprivation, the suppression of ribosomal protein synthesis is reported to correspond with a shift in bacterial metabolism towards survival [35], [36]. This prevents cells from over-investing biosynthetic resources in ribosome synthesis, which is energetically costly [37]. The physiological signals that drive this response in most bacteria are the alarmones phosphorylated guanine nucleotides (pppGpp or ppGpp). This phenomenon is also known as stringent response [38], [39]. Previously, we demonstrated that exudates from N-starved maize contain less amino acids than those from nutrient replete plants [12]. Lower concentration of amino acids in N-deficient maize root exudates may have elicited a stringent response in bacterial cells. Genes that are involved in stringent response have not been characterized in *B. amyloliquefaciens* FZB42. However, genes that are known to be involved in the synthesis of bacterial alarmones in the closely related species *Bacillus subtilis* were not differentially expressed in *B.* *amyloliquefaciens*, such as *relA*, *yjbM*, *ywaC* [40], [41]. In addition, the gene encoding the general stress protein YvyD which is reported to be induced under amino acid starvation was also not differentially expressed in comparison to cells exposed to nutrient sufficient maize exudates [42]. Moreover, in the transient phase, 12 genes encoding ribosomal proteins and two genes involved in membrane bioenergetics such as *qoxD* (respiration) and *atpC* (ATP synthase) were up-regulated (Fig. 3, Table S2). These observations suggest that it is unlikely that the source of stress in the exponential phase was the limited availability of N, given that, at later stages of bacterial growth, nutrients tend to be exhausted in the media. For this reason, if bacterial cells were experiencing amino acid starvation during transient growth, genes involved in protein synthesis would then be expected to be even more repressed rather than induced. Alternatively, the stress could have been caused by the presence of inhibiting compounds in the exudates, such as antimicrobial secondary metabolites. Under conditions of N limitation, roots may release compounds that exhibit antimicrobial activity, such as phytoalexins, which are reported to be released in maize exudates [17], [43]–[47]. It is also possible that antimicrobial compounds may have been present in N-deficient plant root exudates that target the bacterial purine biosynthetic pathway, which was overrepresented in the list of down-regulated genes in FZB42 at OD 1.0 (Table S3). In some PGPR, *purB* influences their rhizosphere colonization ability [48]. As a consequence, competition for N between root-associated bacteria and plants could be prevented by affecting bacterial colonization ability. In this study, however, we did not evaluate antimicrobial properties of root exudates and as such this hypothesis requires further investigation.

Another indication that bacterial cells were under stress when exposed to N-deficient maize root exudates is the overrepresentation of the GO term cell envelope biogenesis/outer membrane in the list of genes up-regulated at OD 1.0. Transcript levels of several stress-related genes as well as alternative, sporulation specific sigma factors SigK and SigG-activating proteins were increased (Fig. 2, Table S3). Different signaling systems known to modulate stress responses have been reported to induce factors involved in the envelope biogenesis and maintenance, including Cpx and SigE [49].

Notably, genes associated with chemotaxis/motility were repressed during the exponential phase (*hag*) and especially in the transient phase (*fliM*, *fliE*, *flgE*, *fliK*, and *fliH*), as evidenced by the overrepresentation of the functional group related to motility and chemotaxis in the list of down-regulated genes at OD 3.0 (Fig. 2 &3, Tables S2 & S3). Bacterial chemotactic motility plays an essential role in root colonization by certain bacteria [50], [51]. Under conditions of N limitation, bacterial motility may be affected by a lower availability of amino acids released in root exudates and/or by plant-derived compounds that inhibit root colonization by repressing bacterial genes associated with chemotaxis and motility to avoid competition for N. In fact, no gene or operon that could improve plant N acquisition (e.g. *nif* genes associated with N fixation) is present in the genome of *B. amyloliquefaciens* FZB42 [52], which suggests that, under conditions of N limitation; these bacteria may compete with roots for scarce N sources. Alternatively, N-deficient plants may not select for bacteria that have no attributes that help improving plant N acquisition, such as FZB42.

### Transcriptional Responses to Exudates from P-deficient Plants in FZB42

A number of chemotaxis and motility-related genes as *flgB*, *flgL*, *flgM*, *fliD*, *fliS*, *fliT*, and *motB* were induced by root exudates of P-starved maize in the transient growth phase (Fig. 3, Table S4). Functional categories associated with motility and flagellum assembly were, therefore, overrepresented in the up-regulated gene list (Table S5). Therefore, we hypothesize that P-starved plants release bacterial chemoattractants in root exudates. Plant uptake of P is often limited in soils due to the low diffusion rates of orthophosphates [53]. As a consequence, microorganisms that are able to mineralize organic P or solubilize inorganic P in soils play a significant role in increasing P availability to plants [54]. *B. amyloliquefaciens* was shown to secrete phytase during transient phase, which is believed to be a key trait associated with maize growth promotion under P limiting conditions [14]. Triggering chemotaxis and motility of phosphate mobilizing bacteria in the rhizosphere may improve root access to sparingly available P. Chemotactic and motility abilities are closely associated with rhizosphere and root colonization, as well as the attraction of specific populations to the root vicinity [55], [56]. Nevertheless, genes involved in motility were down-regulated in the rhizobacterium *Pseudomonas aeruginosa* 7NR when associated with the P-deprived ryegrass (*Lolium perenne*) [57]. However, as opposed to *B. amyloliquefaciens*, so far there is no evidence that *P. aeruginosa* 7NR possess P mobilization capacity. Therefore, similarly to *B. amyloliquefaciens* in conditions of N limitation, plants may prevent competition for P with *P. aeruginosa* 7NR when P is scarce.

Interestingly, we found opposite changes in transcript levels of genes associated with bacterial motility in response to N and P deficient maize root exudates. Bacterial genes related to chemotaxis and motility were down-regulated at OD 3.0 by N-deficient maize root exudates (Fig. 3, Tables S2 and S3). We have previously hypothesized that the lower the mobility of the deficient nutrient, the more primary metabolites are exuded by roots [12]. This may be the case for chemoattractants and therefore have been reflected on transcript levels of chemotaxis/motility related genes in FZB42. P deficiency generates narrower P depletion zones compared to nutrients with higher solubility in the soil, such as nitrate [58]. Therefore, investing resources in exudation and triggering bacterial motility under P deficiency are more likely to benefit the plant due to increased P availability derived from microbial mineralization beyond the P depletion zone. This would not be the case for N as depletion zones for N forms are much wider.

Differences in root exudates between P-deprived and nutrient-replete maize reported in our previous study may provide an indication of which compounds led to an increased expression of bacterial chemotaxis/motility related genes [12]. The metabolite analysis of root exudates collected from P-deprived maize revealed higher concentrations of GABA and several sugars, such as inositol, erythritol, ribitol, fructose, glucose and arabinose in comparison to nutrient-replete plants [12]. As GABA has been associated with signaling in a number of abiotic stress responses [59], [60], it may act as a signaling compound when maize is P-starved. Furthermore, sugars are capable of eliciting chemotaxis responses in bacteria [61], [62]. Therefore, they may also play a role in inducing genes associated with motility in FZB42. Indeed, an enhanced transcription of genes involved in sugar uptake and utilization such as *rbsC*, *rbsD*, *rbsK* and *fruK*, was observed during transient phase of FZB42 cells exposed to P-deficient maize root exudates (Fig. 3, Table S4).

In addition, genes related to cellular responses to stress were down-regulated in the transient phase (Tables S4 and S5). Phosphate deficient plants may, therefore, exude less secondary metabolites that are toxic to bacteria relative to plants cultivated under optimal nutritional conditions. This may occur to attract certain beneficial microbes that can improve acquisition of the specific deficient nutrient. For instance, *B. amyloliquefaciens* stimulates the growth of P-limited maize seedlings in the presence of phytate [14].

### Transcriptional Responses to Exudates from Fe-deficient Plants in FZB42

Exudates from Fe-deficient maize triggered more changes in transcript levels in the transient phase of bacterial growth than at exponential phase (Fig. 1, Table S6). The functional groups related to gene transcription and membrane composition are overrepresented in the lists of up-regulated as well as down-regulated genes, as evidenced by the GO term enrichment analysis (Table S7).

Root exudates collected from Fe-deficient plants induced seven genes that encode transport/binding proteins and lipoproteins in the transient phase (Fig. 3, Table S6). Notably, one of them encodes an ABC transporter for the siderophores Fe-enterobactin and Fe-bacillibactin (*feuB*). This suggests that the Fe-deficient roots may induce the production of siderophores in bacteria. As maize cannot use microbial siderophores directly [63], plants may access the Fe through degradation of these siderophores [64] or via ligand exchange with phytosiderophores [65].

During transient phase, numerous genes involved in sugar uptake and utilization were down-regulated suggesting that only a limited amount of carbohydrates were available from Fe-starved maize root exudates (Table S6). The GO terms glucosidase activity, sporulation, and carbohydrate transport were overrepresented in the down-regulated gene list at this phase (Table S7). Relative to the effects exerted by the exudates collected from plants under N or P deficiency, those from Fe-deficient plant roots did not induce very distinctive changes in the *B. amyloliquefaciens* FZB42 transcriptome.

### Transcriptional Responses to Exudates from K-deficient Plants in FZB42

Only a few bacterial genes were differentially expressed in the exponential phase of bacterial growth in the presence of root exudates collected from K-starved maize. The few regulated genes encode hypothetical proteins with unknown function (Table S8). In the transient phase, 76 genes were repressed, and those genes belonged to various functional groups (Fig. 3). The most significant GO terms that were enriched in this gene list (*P*<0.05) were the cell wall macromolecule catabolic process and oxidation-reduction (Table S9). In bacteria and eukaryotic cells, K is a key intracellular cation, which is largely associated with osmoregulation [66], but also to the maintenance of enzyme functions [67].

### Most Discriminating Genes Between Treatments

**Figure 4:**
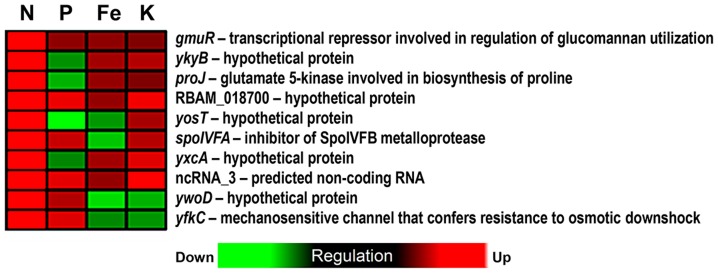
Discrimination of bacterial transcriptional responses to root exudates collected under nitrogen (N), phosphate (P), iron (Fe), and potassium (K) deficiency treatments. The first two axes of the Between Group Analysis using Canonical Analysis (BGA-CA) are shown. Biological replicates corresponding to the deficiency treatments were represented by an ellipse. The ten most discriminating genes for each treatment are labeled on the corresponding coordinates which indicate their discriminative power according to the BGA. The designations for non-coding RNAs ncRNA_2 and ncRNA_6 corresponds to gene IDs FZB42_3912 and FZB42_4030, respectively.

**Figure 5:**
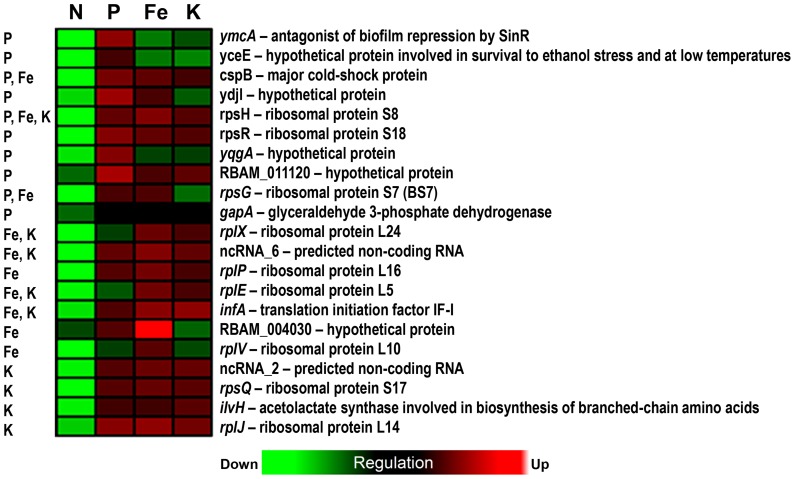
Heat map showing transcript levels of the most discriminating bacterial genes for nitrogen (N), phosphorus (P), iron (Fe), and potassium (K) deficiency exudate treatments. Designations of treatments on the left of the heat maps (N, P, Fe, or K) correspond to treatments which the described gene is discriminating for. The designations for non-coding RNAs ncRNA_3, ncRNA_6, and ncRNA_2 correspond to gene IDs FZB42_3984, FZB42_4030, and FZB42_3912, respectively.

Deficiency treatments could explain most of the variation between bacterial transcriptional profiles only in the exponential growth phase (BGA-CA, *P*<0.05), but not in transient growth phase (BGA-CA, *P*>0.05). The reduced number of differentially expressed genes is likely to better reflect responses to specific deficiencies as opposed to later stages of the growth, when responses may overlap due to secondary bacterial responses to root exudates. Similarly to the present study, previous investigations conducted on the effect of root exudates on transcript levels of *B. amyloliquefaciens* also found that more genes were differentially expressed at the transition to stationary growth phase (OD 3.0) compared to the exponential growth phase (OD 1.0) [20]. The most evident trend shown by the BGA-CA was the separation of transcriptional profiles of cells exposed to exudates from the N-deficiency treatment from profiles associated with cells exposed to the other exudates (Fig. 4). The N deficiency treatment was associated with ten up-regulated genes that were not differentially expressed in the other treatments (Fig. 5). Four of these genes are involved in the regulation of glucomannan utilization (*gmuR*), biosynthesis of proline (*proJ*), resistance to osmotic downshock (*yfkC*) and control of SigK (sporulation-specific sigma factor) (*spoIVFA*). The others transcribe regulatory RNAs (see next section) and hypothetical proteins with unknown function. Proline is implicated as a stress protectant in plants and bacteria [68], being mostly associated with adaptation to osmotic stress in the later [69], [70]. Additionally, another gene associated with N deficiency treatment encodes a mechanosensitive channel (*yfkC*) involved in resistance to osmotic downshock (exposure to hypo-osmotic environments). It is possible that a common stress-associated regulator may have affected the expression of those genes as well. SigB represents a potential candidate for such a function, since it is a general stress regulator that also regulates other MscS-type putative channel-forming proteins (YkuT) [71].

As evidenced by the overlap between ellipses along BGA1, the transcriptional responses to P, Fe, and K starved maize root exudates were more similar to each other and shared some of the most discriminating genes. Except for one under P and one under Fe deficiency, the transcript levels of the other genes were not considerably altered in P, Fe, and K, but were mostly down-regulated in N-deficiency treatment (Fig. 5). Most of them encoded ribosomal proteins involved in translation. Others included an antagonist of biofilm repression involved in regulation of biofilm formation (*ymcA*), a hypothetical protein associated with survival to ethanol stress and at low temperatures (*yceE*), a major cold-shock protein involved in RNA chaperone activity (*cspB*), a catabolic enzyme in glycolysis (*gapA*), and an acetolactate synthase involved in the biosynthesis of branched-chain amino acids (*ilvH*). In some human pathogens including *Neisseria meningitidis*, the *gapA*-encoded enzyme glyceraldehyde 3-phosphate dehydrogenase plays a role in colonization and invasion of host tissues [72]. However, its role in plant-microbe interactions still remains to be elucidated.

### Non-coding RNA Genes

Bacterial non-coding RNAs also referred as small regulatory RNAs, ‘sRNAs’, generally modulate changes in cellular metabolism in response to environmental changes, especially under suboptimal or stressful growth conditions [73], [74]. Sixty-three sRNAs are indicated as ‘BSU-misc_RNAs’ within the genome of *B. subtilis* 168 (NC000964). The number of potential sRNAs in *B. subtilis* has been recently increased to upwards of 100 candidates [75]. sRNAs have not been experimentally studied before in *B. amyloliquefaciens*, but a comparative genome-based screen has been performed previously, yielding 238 candidate genes within the genome of FZB42 [24] (Table S10). Thirty-eight small non-coding RNAs had altered transcription by FZB42 when exposed to different maize root exudates. Most of them were found affected in their expression when FZB42 was exposed to root exudates obtained from N-starved maize plants (Table 2). The sRNAs FZB42_3931, corresponding to BSU_misc_RNA_47, and FZB42_4026, a possible TPP riboswitch corresponding to BSU_misc_RNA_4, were experimentally confirmed by Northern blot hybridization (B. Fan, unpublished results).

**Table 2:** Expression of small regulatory RNA genes in FZB42 cultivated in presence of maize root exudates extracted from nutrient starved maize plants (N, nitrogen; P, phosphorous, Fe, iron, K, potassium).

Gene ID	prev	next			N		P		Fe		K	
	gene	gene	from	to	1.0	3.0	1.0	3.0	1.0	3.0	1.0	3.0
FZB42_4040	*rpmH*	*dnaA*	214	283		−1,91						
FZB42_4038	*dnaA*	*dnaN*	1793	1862		−2,26						
FZB42_3908	*gyrA*	*16SrRNA*	9608	9677		−4,09		−3,22				
FZB42_3909	*bofA*	*16SrRNA*	31089	31158		−1,91						
FZB42_3910	*lysS*	*16SrRNA*	90940	91009		−3,59		−1,37				
FZB42_4034	*5SrRNA*	*ctsR*	102078	102147							1,81	
FZB42_3840	*nusG*	*rplK*	119195	119264	−2,07							
FZB42_4030	*ybxF*	*rpsL*	130350	130399	−2,69							
FZB42_4026	*truA*	*rplM*	154480	154549	−2,31							
FZB42_3911	*ybaN*	*16SrRNA*	161480	161549		−2,63						
FZB42_3832	*hyp*	*cspC*	521322	521391		−2,06						
FZB42_4018	*ydeB*	*yrkD*	522856	522925	2,58							
FZB42_3912	*ydjK*	*tRNA_Arg*	604598	604647							1,9	
FZB42_4014	*ydiL*	*groES*	619377	619446		−7,2				−7,53		
FZB42_3830	*hyp*	*pbuG*	656536	656605	2,2			−4,89				−3,26
FZB42_3834	*ygaJ*	*thiC*	885381	885312	1,74							
FZB42_4007	*cspB*	*yhcJ*	914858	914789	−1,87							
FZB42_3878	*hyp*	*yjbH*	1134155	1134224	−2,1							
FZB42_3875	*ykoX*	*ykoY*	1283138	1283207								6,16
FZB42_3992	*defB*	*ykyA*	1390339	1390408	1,85		2,06					
FZB42_3984	*hyp*	*ylxM*	1587076	1587145	2,01						1,99	
FZB42_3982	*ffH*	*rpsP*	1588909	1588958	−2,06							
FZB42_3805	*yoaD*	*yoaE*	2001779	2001710			2,88					
FZB42_3976	*yobL*	*csaA*	2008166	2008235		2,58				4,26		
FZB42_3873	*panC*	*birA*	2160078	2160147		−1,89				−2,78		−3,96
FZB42_3855	*yqeN*	*comEC*	2506968	2506899	1,79							
FZB42_3947	*yrvM*	*aspS*	2585300	2585231					1,84			
FZB42_3943	*nadB*	*nifS*	2614025	2613976							1,89	
FZB42_3941	*rplU*	*spoIVFB*	2620441	2620372	−2,06							
FZB42_3940	*valS*	*ysxE*	2633842	2633911	2,08				1,77			
FZB42_3931	*infC*	*ysbB*	2716865	2716796	−1,81							
FZB42_3925	*yjdF*	*ytwI*	2746197	2746128		−3,1						
FZB42_3849	*tyrS*	*acsA*	2805683	2805614		−1,95				−2,02		
FZB42_3905	*16SrRNA*	*yuaJ*	2928068	2927999	−1,86							
FZB42_3900	*yvbW*	*hyp*	3253811	3253880	1,76							
FZB42_3895	*yvcI*	*trxB*	3315101	3315032		−2,05				−1,85		
FZB42_3891	*ywbE*	*hyp*	3661578	3661529						1,82		
FZB42_3843	*rpsF*	*engD*	3902898	3902849	−2,42							

Fold change (FC) in comparison to the control (exudate from maize plants grown without nutrient limitation) is indicated.

Detecting regulatory pathways in which specific non-coding RNAs are involved was not within the scope of this study, but these findings may be used as an initial reference for detecting small RNAs modulating bacterial responses to plant nutritional deficiencies.

### Correlation Between Bacterial Gene Expression and Metabolite Composition of Root Exudates

**Figure 6:**
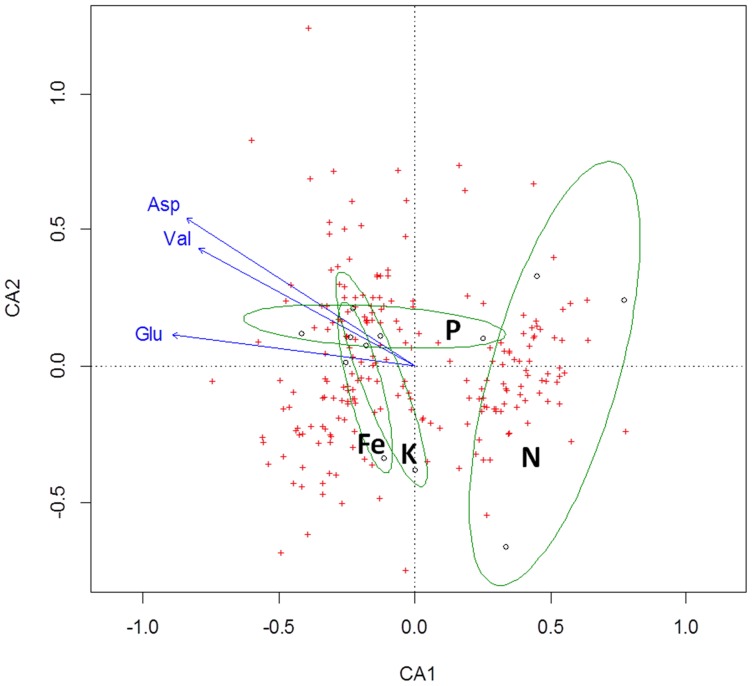
Canonical Analysis (CA) of bacterial transcript levels and vector fitting of the root exudate metabolites. Blue arrows represent significant fittings (P<0.05). Green ellipses depict biological replicates of nutritional deficiency treatments applied to maize plants from which root exudates were collected. N = Nitrogen deficiency, P = Phosphate deficiency, Fe = Iron deficiency, and K = Potassium deficiency. Three small circles located inside each ellipse represent biological replicates for each treatment. Red crosses represent gene coordinates.

Vector fitting was applied to identify whether changes in the composition of root exudates significantly correlated with deficiency treatments and genes. The compounds in root exudates that showed significant correlation with the ordination were the amino acids aspartate (Asp), valine (Val) and glutamate (Glu) (*P*<0.05) (Fig. 6). Therefore, from all 29 measured dominant metabolites in root exudates, only three amino acids were significantly correlated with bacterial transcriptome changes associated with exudates from different nutrient deficiency treatments. This observation suggests that overall changes in the bacterial transcriptome could not be correlated to most of the measured dominant metabolites. By observing the direction of the arrows that illustrate changes in the concentration of individual compounds, it is notable that these compounds mostly explain a separation along the first axis of the CA (CA1) (Fig. 6). This axis, as also observed in the BGA-CA (Fig. 4), separated primarily the N deficiency from P, Fe and K deficiency treatments. Since the N-deficient maize root exudates had a lower concentration of amino acids [12], this observation suggests that changes in transcriptional profiles may be partially attributed to differences in quantities of Asp, Val and Glu between treatments.

The expression of the most discriminating genes may be associated with decreases in metabolite concentrations in root exudates. There were two major trends. One was the repression of genes involved in translation (ribosomal proteins), biosynthesis of branched chain amino acids (*ilvH*) and response to ethanol stress (*yceE*) by N-deficient maize root exudates. The other was the induction of genes associated with the control of sporulation (*spoIVFA*) and biosynthesis of proline (*proJ*) (Fig. 5). Beside the fact that branched-chain amino acids constitute the majority of amino acids in proteins, they play a special role in *Bacillus* spp. as they act as precursors of major fatty acids of membrane lipids [76]. The biosynthesis of these amino acids have also been described as crucial for the ability of beta-rhizobia to grow in a free living state and to form symbiosis with plants [77]. Enzymes of the branched-chain amino acids biosynthetic pathway, such as acetolactate synthase encoded by the gene *ilvH*, can be potential target of inhibiting compounds present in N-deficient maize exudates. For example, certain herbicides inhibit this enzyme in plants [78]. Amino acids have been also reported to serve as signaling molecules for microorganisms [79]. They function as communication molecules in the initiation of fruiting body formation in soil-dwelling microorganism *Myxococcus xanthus* [80], [81], and particularly glutamate and aspartate play a role during autoaggregation in chemotactic *Escherichia coli* [82]. Another study that performed a genome-wide analysis of *B. subtilis* transcriptional responses induced by glutamate, valine and glutamine pulses revealed that the metabolism of the bacteria was reprogrammed and showed both similarities and dissimilarities between amino acid pulses [83]. However, interpretation over the expression of thousands of genes based on 29 dominant primary metabolites present in a complex mixture of various compounds like root exudates has to be made with caution. Anyways, such observations can provide new insights to start understanding complicated systems such as molecular plant-microbe interactions. A number of 120 compounds in maize root exudates were assessed through GC-MS to investigate differences in metabolite profiling of distinct genetically modified maize [84]. In the present study, only the most dominant primary metabolites in root exudates were considered. However, secondary metabolites released by roots such as flavonoids, strigolactones, and benzoxazinoids [85]–[87] are typically associated with signaling in plant-microbe interactions. Due to the ubiquity of primary metabolites in soils, other compounds rather than amino acids, organic acids and sugars are better candidates to act as signals for nutrient starvation in plants. Ideally, a comprehensive investigation should include all detectable metabolites (both primary and secondary) by a sensitive method [88] and a multivariate statistical approach may be best suited to correlate metabolites with transcriptional profiles and detect the most important compounds that trigger gene expression.

### Conclusions

**Figure 7:**
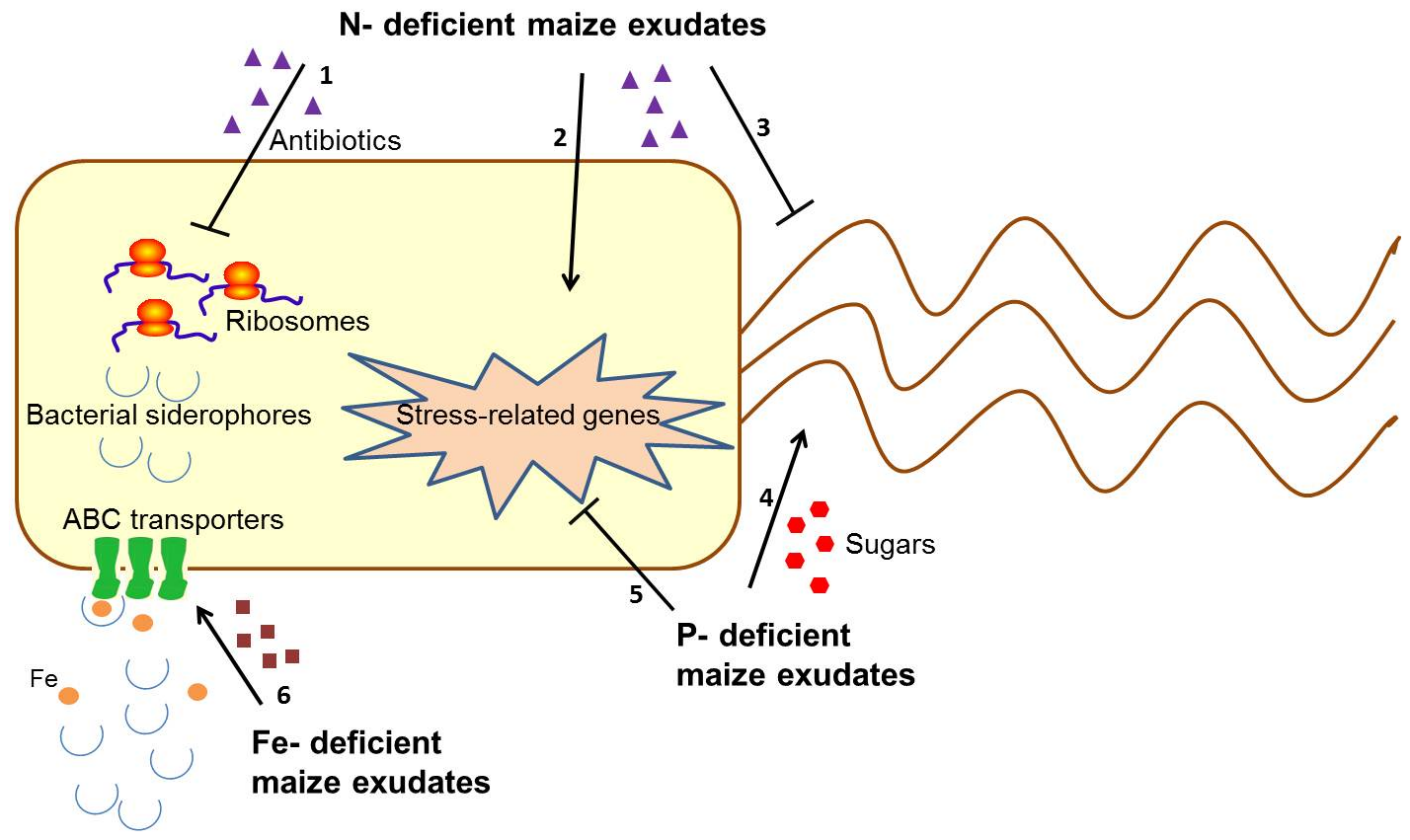
Schematic drawing of a bacterial cell showing the most prominent hypothesized functions altered by N, P, and Fe-deficient maize exudates. 1. Repression of ribosomal proteins by N-deficient maize exudates (possibly antibiotic compounds); 2. Induction of stress-related genes by N-deficient maize exudates; 3. Repression of genes involved in bacterial motility by N-deficient maize exudates; 4. Induction of genes involved in bacterial motility by P-deficient maize exudates (most likely by sugars). 5. Repression of stress-related genes by P-deficient maize exudates; 6. Induction of an ABC transporter for bacterial siderophores by Fe-deficient maize exudates.

In this study, we provide evidence to suggest that the nutritional status of maize plants affects the transcriptome of a beneficial root colonizing bacterium due to changes in composition of root exudates. The main hypothesized bacterial functions are schematically depicted in Figure 7. Exudates from N-starved maize trigger the most drastic overall changes in the transcriptome of *B. amyloliquefaciens* in the exponential growth phase compared to P, Fe and K. Many of the changes were caused by the repression of genes associated with protein synthesis. Genes associated with chemotaxis and motility were down-regulated by N-deficient maize exudates, but up-regulated by the P-deficient maize exudates. These observations indicate that, at least in the case of maize and *B. amyloliquefaciens*, the nutritional status of plants influences the physiology of associative bacteria for their own benefit. As there is no evidence that *B. amyloliquefaciens* enhance plant N acquisition, it is possible that under conditions of N deprivation, in which bacteria can compete for N, bacterial activities may be suppressed. However, when P is limiting, plants may attract microbes that exhibit attributes that increase P acquisition, such as the PhoP/PhoR dependent production of phytase, which is an attribute possessed by *B. amyloliquefaciens* [89]. It also appears that chemotactic responses towards carbohydrates in root exudates play an important role in the interaction between P-deficient maize and *B. amyloliquefaciens*. Furthermore, Fe-deficient maize exudates induced an ABC transporter for bacterial siderophores in FZB42, which may increase Fe availability to plants. These hypotheses warrant further testing using soil instead of nutrient solution as a substrate for plant cultivation [90], as well as different PGPR-plant associations, or even culture-independent approaches [91]. The identification of metabolites that affected differences in the bacterial transcriptome shed light on molecular communication patterns utilized by maize to better cope with stressful environmental conditions such as shortage of nutrients.

## Supporting Information

Table S1 **Sequences of primers used in the quantitative real-time PCR.**
(DOCX)

Table S2 **Differentially expressed bacterial genes in the presence of nitrogen-deficient maize root exudates in exponential (OD 1.0) and transient growth phase (OD 3.0).**
(XLSX)

Table S3 **Summary of ‘the functional annotation clustering’ tool analysis for the nitrogen deficiency treatment exudates on bacterial gene expression.**
(XLSX)

Table S4 **Differentially expressed bacterial genes in the presence of phosphate-deficient maize root exudates in exponential (OD 1.0) and transient growth phase (OD 3.0).**
(XLSX)

Table S5 **Summary of ‘the functional annotation clustering’ tool analysis for the phosphate deficiency treatment exudates on bacterial gene expression.**
(XLSX)

Table S6 **Differentially expressed bacterial genes in the presence of iron-deficient maize root exudates in exponential (OD 1.0) and transient growth phase (OD 3.0).**
(XLSX)

Table S7 **Summary of ‘the functional annotation clustering’ tool analysis for the iron deficiency treatment exudates on bacterial gene expression.**
(XLSX)

Table S8 **Differentially expressed bacterial genes in the presence of potassium-deficient maize root exudates in exponential (OD 1.0) and transient growth phase (OD 3.0).**
(XLSX)

Table S9 **Summary of ‘the functional annotation clustering’ tool analysis for the potassium deficiency treatment exudates on bacterial gene expression.**
(XLSX)

Table S10 **List of candidate regulatory RNAs in** ***B. amyloliquefaciens*** **FZB42.**
(XLSX)
