# Supplementary material for: Linking Plant Nutritional Status to Plant-Microbe Interactions
Source: PLoS One. 2013 Jul 16;8(7):e68555. doi: 10.1371/journal.pone.0068555 (PMC3713015; doi:10.1371/journal.pone.0068555)
Supplement: Table S1 — Sequences of primers used in the quantitative real-time PCR. (DOCX) [file pone.0068555.s001.docx]

**Suppl. Table S1.** Sequences of primers used in the quantitative real-time PCR

| Target transcript | Forward Primer (5’ -> 3’) | Reverse Primer (5’ -> 3’) |
| --- | --- | --- |
| *licH* | CCCGGCAAATTGCACTTC | TGGGCGTCGCAAAAGC |
| *iolC* | GCGGACGGTTTCATCGTTAC | CGCGTGACGAATTTGACGTA |
| *yvqH* | CATTTCAGTCAGCGCCTTTTT | CAGGCTCAGCTTGCTTTCG |
| *dhaS* | CCGGCCTTCACCAAGATACA | AAAGCACGCGCTCATGCT |
| *licB* | TCCGCTCCGTTGCATGT | GGAGTGCCGGTTGAAGTCA |
| *flgL* | CGATTTCTGTACCGATTGCTTTC | TCCGGGACTTGATGGTTCA |
| *fliS* | TTCATTTTTGGCTTCAAGGTTGT | CTGCCTGCGATTCATTAAGCT |
| *iolH* | CGTGGTCCGGCTTGATTC | GCGACGAACGCCGTTTT |
| *yocH* | CAACACCGGTTGCCGTTAC | TGACTGCAACTGCTTACTCTGCTA |
| *clpC* | GCGATTGACGCGTCGAATA | CGCGCCGATGCATTG |
| *glmS* | GCGCTGGCGACACAAGA | GCTGCGACTTCCTTCACGTT |
